# Synthesis, Characterization, and Magnetocaloric Properties of the Ternary Boride Fe_2_AlB_2_ for Caloric Applications

**DOI:** 10.3390/ma17163886

**Published:** 2024-08-06

**Authors:** Vaibhav Sharma, Radhika Barua

**Affiliations:** Department of Mechanical & Nuclear Engineering, Virginia Commonwealth University, 401 West Main Street, Richmond, VA 23284, USA; sharmav23@vcu.edu

**Keywords:** magnetocaloric effect, MAB phases, Fe_2_AlB_2_, review, ordered atomic structure, heat pumping, transport properties, mechanical and electrical properties, sustainable manufacturing, additive manufacturing

## Abstract

The ternary transition metal boride Fe_2_AlB_2_ is a unique ferromagnetic “MAB” phase that demonstrates a sizable magnetocaloric effect near room temperature—a feature that renders this material suitable for magnetic heat pump devices (MHP), a promising alternative to conventional vapor compression technology. Here, we provide a comprehensive review of the material properties of Fe_2_AlB_2_ (magnetofunctional response, transport properties, and mechanical stability) and discuss alloy synthesis from the perspective of shaping these materials as porous active magnetic regenerators in MHPs. Salient aspects of the coupled magnetic and structural phase transitions are critically assessed to elucidate the fundamental origin of the functional response. The goal is to provide insight into strategies to tune the magnetofunctional response via elemental substitution and microstructure optimization. Finally, outstanding challenges that reduce the commercial viability of Fe_2_AlB_2_ are discussed, and opportunities for further developments in this field are identified.

## 1. Introduction

The MAB phases are a fascinating class of materials within transition metal borides (TMBs) that are distinguished by their unique atomic layering and complex stoichiometry, described by the general formula (MB)_2z_A_x_(MB_2_)_y_, where z = 1–2, x = 1–2 and y = 0–2 [1]. All MAB phases exhibit an ordered atomic structure with alternating layers of transition metal–boron sublattices and interleaving A-atom layers—a distinctive layering that impacts the material properties significantly, including electrical conductivity, thermal stability, and mechanical strength. In recent years, Al-containing MAB phases bearing the chemical formula MAlB, M_2_AlB_2_, M_3_Al_2_B_2_, M_3_AlB_4_, and M_4_AlB_6 (_M = transition element) have garnered significant attention in the scientific community, particularly since computational studies indicate the possibility of etching the Al atoms from MAB phases, thus forming 2D MXenes multilayer nanosheets that demonstrate excellent potential for use in energy storage and electrocatalysis. Experimentally, only MAlB (M = Mo and W), M_2_AlB_2_ (M = Cr, Mn, and Fe), Ru_3_Al_2_B_2_, Cr_3_AlB_4_, and Cr_4_AlB_6_ have already been synthesized to date. Among these, Fe_2_AlB_2_ stands out as a stable ferromagnet with a magnetic ordering temperature of around 300 K, which is considered promising for magnetic heat pumping (MHP). This solid-state technology may be used for various applications in the energy sector, ranging from building cooling/heating and water heating to low-grade waste heat recovery.

MHP relies on the magnetocaloric effect (MCE)—a magnetothermodynamic phenomenon wherein a magnetic field is varied in a cyclic adiabatic manner near a given material’s magnetic ordering temperature, resulting in a significant change in magnetic entropy (ΔS_mag_) and temperature (ΔT_ad_). This effect can be harnessed through a Brayton-based active magnetic regenerator (AMR) thermodynamic cycle, where magnetic fields are applied to and removed from a magnetocaloric material (MCM) in step with an alternating flow of water-based heat transfer fluid to pump heat over temperature spans larger than the temperature change in the material. Beyond the strength of the magnetocaloric response (large ΔT_ad_ in moderate magnetic fields), the choice of MCMs for AMR devices also depends on several additional factors: (1) good heat transfer characteristics (low value of the heat capacity C_p_ and high thermal conductivity κ) to facilitate efficient convective heat transfer from or to the working fluid, (2) high electrical resistivity to reduce eddy current losses during magnetic field cycling (3) robust mechanical and chemical stability upon prolonged exposure to the heat transfer fluid, and finally (4) ease of manufacturing into porous energy-efficient heat exchange structures. Over the last three decades, several intermetallic compounds that demonstrate an appreciable room-temperature MCE have been reported for building heat/cooling MHPs, including Gd_5_Si_2_Ge_2_, La(Fe, Si)_13_ alloys and their hydrides, MnFe(P, Ge, Si, As) pnictides and NiMn-based Heuslers alloys. Since Shatruk and co-workers discovered MCE in Fe_2_AlB_2_ in 2013, there has also been a sustained increase in published research on this unique MAB compound [2].

Building on this body of work, this topical review aims to provide a synopsis of the material properties of Fe_2_AlB_2_, specifically in the context of their potential use in MHPs. Following a discussion regarding the phase relationships in the Al-Fe-B phase diagram and its implications in materials synthesis, salient aspects of the coupled magnetic and structural phase transitions in Fe_2_AlB_2_ are outlined to elucidate the fundamental origin of the MCE observed in this compound. Next, strategies to tune the magnetocaloric response of Fe_2_AlB_2_ via elemental substitution are presented. After that, the heat transfer characteristics, electrical transport properties, mechanical properties, and chemical stability of the parent compound and its compositional variants are summarized. Last, from the perspective of manufacturing costs and energy efficiency, the ease of fabrication of magnetic regenerators with appropriate architectures for MHP devices using Fe_2_AlB_2_ will be discussed. Finally, a comprehensive comparison of Fe_2_AlB_2_ material properties and other promising MCMs will be provided to gain insight into the prospects of using magnetic MAB compounds for magnetocaloric applications. 

## 2. Fundamentals

**(a) Phase Diagrams: Key to Optimal Materials Processing**

**Figure 1:**
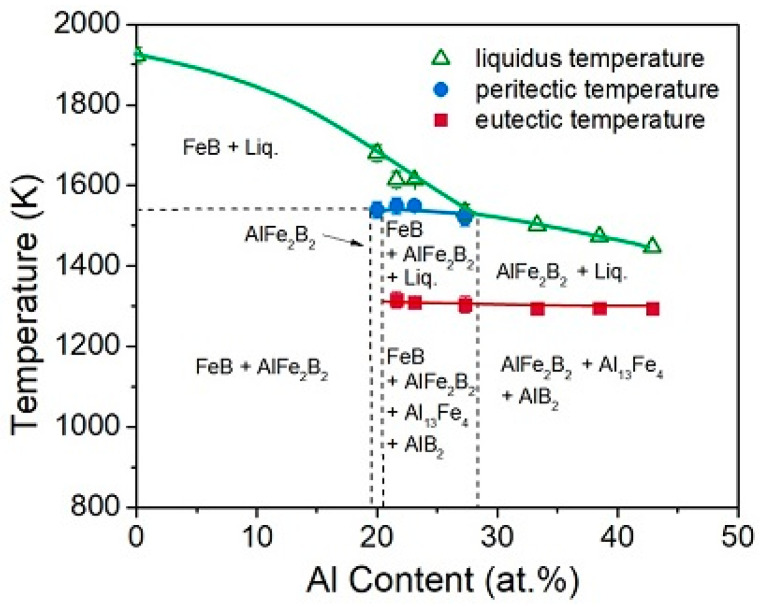
Fe_2_AlB_2_ pseudo-binary phase diagram showing the liquidus, peritectic, and eutectic temperatures of polycrystalline samples. Figure taken from [4].

Understanding the phase diagram Fe_2_AlB_2_ is crucial in processing because it provides valuable information about the relationships between temperature, composition, and the phases present in the material [3]. Insights, thus obtained, help predict how the alloy will behave under different conditions and how they can be manipulated to achieve desired properties. To this end, the pseudo-binary Al-FeB phase diagram, shown in Figure 1 [4], reveals the experimentally determined liquidus, peritectic, and eutectic temperatures of polycrystalline samples of nominal Al:Fe: B composition x:2:2, where x ranges from 1 to 3. In the 3:2:2 composition, the liquidus temperature decreases with increasing Al content from 1923 K (the melting point of binary FeB) to 1447 K. The peritectic temperature remains nearly constant at ~1540 K until the peritectic and liquidus lines intersect at ~28 at.% Al, indicating the limit of FeB stability. The eutectic temperature remains nearly constant at ~1300 K, forming a eutectic solid–liquid boundary extending into the phase diagram’s Al-rich region [4]. 

Phase diagrams are crucial in materials processing because they map the stability of different phases (solid, liquid, gas) and their compositions at various temperatures and pressures. Understanding these diagrams helps predict how materials will behave under different conditions and how they can be manipulated to achieve desired properties. Following these guidelines, it is deduced that when Fe_2_AlB_2_ is formed from melt processes (arc-melting, suction-casting, direct energy deposition (DED) additive manufacturing), the Fe_2_AlB_2_ compound forms by a peritectic reaction with a limited range of solid solutions ranging from 20.5 < Al at.% < 19.5. 

Formation of secondary phases (FeB, AlB_2_, and Al_13_Fe_4_) cannot be avoided when the alloy solidifies through this two-phase region [2,5,6,7,8,9,10,11,12,13,14,15,16,17,18], which extends over 200 °C at the cooling rates available during standard melt-based processing, and typically, the alloy is annealed below the peritectic temperature to improve its phase purity further. Since Al has a low melting point of ~660 °C, excess Al (~20–50 mol% above stoichiometry) is often added to compensate for its evaporation. While excess Al can inhibit the formation of the FeB phase, it leads to Al-rich secondary impurities (namely Al_13_Fe_4_), which are subsequently removed via etching in dilute acid solutions [11].

Challenges associated with melt-based processes may be overcome using solid-state reaction routes (spark plasma sintering, microwave-assisted sintering, reactive hot pressing, molten salt shielding sintering) wherein precursor powder mixtures are pressed, consolidated and heat-treated to achieve dense samples that are primarily single-phase. Following the phase diagram, it is understood that such reactions typically begin with Al combining with Fe to form the FeAl_6_ phase: Fe + 6Al → FeAl_6_. Subsequently, Fe reacts with B to form the FeB phase: Fe + B → FeB, and eventually, the Fe_2_AlB_2_ phase is formed via the FeAl_6_ + 11 FeB + B → 6 Fe_2_AlB_2_ reaction [19]. 

For comprehensive reference, Table S1 in the Supplementary Material lists various melt-solidification and solid-state reaction methods used to produce Fe_2_AlB_2,_ together with relevant information regarding the stoichiometry of the constituent elements and critical processing conditions (heat treatment time, temperature, and environment) [2,3,5,6,7,8,9,10,11,12,13,14,15,16,17,19,20]. Solid-state sintering reactions typically occur at lower temperatures than melt processing, leading to significant energy savings. Lower temperatures reduce thermal stresses and potential distortion or cracking in the material. The fine-grained and homogeneous microstructure obtained through reaction sintering results in superior mechanical properties relative to samples formed via melt processing, resulting in increased strength and hardness. Among the solid-state processing routes, the molten salt shielded sintering (MS^3^) technique stands out as it uses non-reactive molten salts (NaCl/KBr) to protect the precursor powders from oxidation during high-temperature processing in the open air; the use of molten salt further reduces the synthesis temperature, resulting in additional cost savings [19]. The MS^3^ process can be scaled up to an industrial scale for the production of magnetic regenerators by simply increasing the batch size or making the process continuous, and it is recognized as a sustainable manufacturing process because the salts used are not volatile or harmful and can be potentially recycled. 

**(b) Coupled Structural and Magnetic Properties**

In MCMs, induced temperature changes due to the application of a magnetic field are a direct consequence of the interplay between the magnetic moments and the atomic lattice (phonons). At constant pressure, the total entropy of a magnetic substance is a function of both magnetic field (H) and temperature. It consists of magnetic (ΔS_mag_), lattice (ΔS_lat_), and electronic (ΔS_el_) contributions and can be expressed as follows:

ΔS(T,H) = ΔS_mag_(T,H) + ΔS_lat_(T,H) + ΔS_el_(T,H) (1)

**Figure 2:**
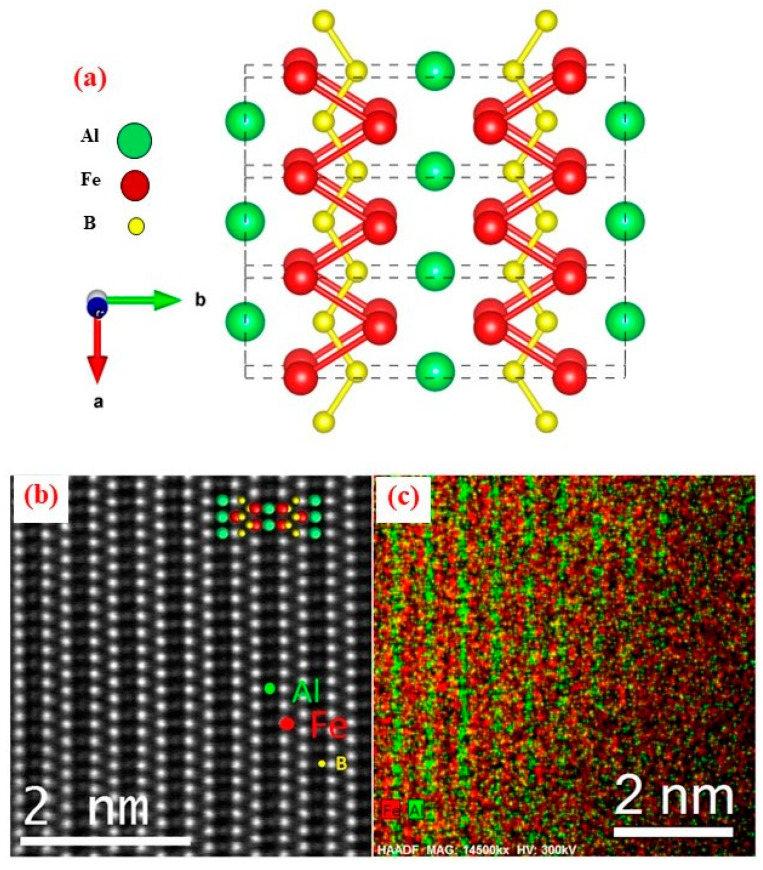
(**a**) Crystallizing in the space group *Cmmm*, the orthorhombic Fe_2_AlB_2_ structure consists of layers of trigonal prisms within the (*ac*) plane formed by Fe atoms surrounding central B atoms. These trigonal prismatic layers are stacked along the *b*-axis and separated by layers of Al atoms. (**b**) High-resolution high-angle annular dark-field imaging (HAADF) scanning transmission electron microscopy (STEM) image of Fe_2_AlB_2_ taken along the [101] zone axis, accompanied by a projection of the unit cell marked with Fe (red), Al (green), and B (yellow) spheres. The arrangement of Al and FeB slab layers within the unit cell, as depicted in panel (**a**), is also observable; (**c**) EDS elemental mapping excluding the scattering effect of B, showing green stripes for Al and red stripes for Fe distributions. Figure adapted from Ref. [9].

In general, the magnetic field dependence of S_Lat_ and S_el_ is negligible, while S_mag_ is very sensitive to the external magnetic field. When the magnetic field is isothermally applied, the magnetic moment’s arrangement is reorganized, enhancing or reducing the S_mag_, depending on the material’s initial magnetic state. Applying the magnetic field in ferromagnets tends to orient the magnetic moments along the field direction (see Figure 2), making the magnetic material more ordered. This decreases the magnetic entropy and consequently the total entropy by:

ΔS(T,H_Initial → Final_) = S_F_(T,H_Final_) − S_I_(T,H_Initial_) (2)

with H_Final_ > H_Initial_ and S_Final_ < S_Initial_. The full entropy is conserved in adiabatic conditions, i.e., S_F_(T,H_Final_) = S_I_(T,H_Initial_). 

Consequently, the loss in S_mag_ (T,H) is compensated by the change in the quantity S_lat_ (T,H) _+_ S_el_ (T,H) in the opposite way, increasing then the material temperature, ΔT_ad_. 

The quantities ΔT_ad_ and ΔS_mag_ are amongst the most used figures of merit to identify the potential of magnetocaloric materials. Both quantities are related to the temperature change in magnetization, M, and specific heat, Cp:

(3) $$∆T_{ad}=-\int_{H_{o}}^{H_{1}}{\left(\frac{T}{C\left(T,H\right)}\right)}_{H}{{\left(\frac{\partial M\left(T,H\right)}{\partial T}\right)}_{H}dH}_{.}$$

(4) $${\Delta S}_{mag}\left(H,T\right)={µ}_{0}\int_{0}^{H_{max}}{\left(\frac{\partial M}{\partial T}\right)}_{H}dH$$

where μ_0_ is the permeability of free space, H_max_ is the maximum applied field, and T is the temperature. Combining Equations (3) and (4), the relationship between the two quantities can be expressed as:

(5) $$∆T_{ad}\approx -\frac{T\Delta Sm}{Cp}$$

The MCE is most pronounced close to a magnetic transition temperature, and its features depend on the thermodynamic character of the magnetic transformation itself. Discontinuous first-order magnetic phase transitions (FOMPTs) are commonly observed when magnetic and crystallographic phase transformations occur in tandem in MCMs due to the strong interplay between the spins and orbitals of the magnetic moment-carrying species and the underlying crystal lattice. MCMs with FOMPTs demonstrate a change in latent heat and usually exhibit a large, although sharp, isothermal entropy change near their magnetic transition temperatures compared to materials with second-order phase transitions (SOPT) wherein the MCM merely undergoes a change in magnetic order with no accompanying structural transformation or latent heat evolution. Against this background, it is critical to correlate the lattice character of Fe_2_AlB_2_ with magnetic response as it provides invaluable insight into the fundamental drivers that control and fine-tune magnetic properties for reliable operation in real-world MHP applications. 

**Figure 3:**
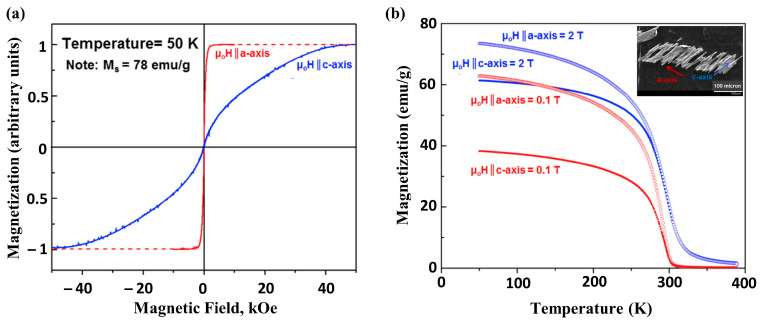
(**a**) Field-dependent magnetization curves of a single Fe_2_AlB_2_ crystallite at 50K. For all magnetic measurements, the external magnetic field was applied in the (ac) plane, either along the *a*- or the *c*-axis of the orthorhombic crystal structure. (**b**) Temperature-dependent magnetization curves of the Fe_2_AlB_2_ crystallite, obtained at an applied field (μ_0_H_app_) of 0.1 T and 2 T; Figure taken from Ref. [17].

In its equilibrium state, Fe_2_AlB_2_ crystallizes in the orthorhombic Mn_2_AlB_2_-type structure that consists of two formula units wherein Fe and B atoms form corrugated Fe_2_B_2_ layers within the *ac* plane that are connected through an Al-atom spacer layer along the *b*-axis, as shown in Figure 2. Within the [Fe_2_B_2_] slab, the B atoms form infinite B–B zigzag chains along the *a*-axis, with Fe-B bonds stitching these chains into the two-dimensional structure, as shown in Figure 2b,c. Near room temperature, Fe_2_AlB_2_ is ferromagnetic in character with reported magnetic moments in the range 0.95–1.32 μB/Fe-atom [2,3,5,8,15]. Both ab initio calculations and experimental studies reveal that the magnetic moments in the Fe_2_AlB_2_ structure are oriented along the crystallographic *a*-axis [9]. The *a-* and *c*-axes of Fe_2_AlB_2_ are identified as the easy and hard axes of magnetization, respectively, with an in-plane anisotropy energy of *K*∼1 MJ/m^3^, Figure 3a [17]. Upon heating, the compound undergoes a ferromagnetic (FM) to paramagnetic (PM) transition with reported Curie temperature *T_c_* values ranging from 282 K to 320 K, as shown in Figure 3b. Application of an external magnetic field shifts T_c_ to higher values at a rate of ~dT_c_/dH = 2 K/T, temperature-dependent resistivity measurements under hydrostatic pressure show that transition temperature *T*c is suppressed down to 255 K for *p* = 2.24 GPa pressure with a dT_c_/dP of ∼−8.9 K/GPa. A simple linear approximation suggests that around 31 GPa would be needed to stabilize the FM phase and suppress the T_c_ completely [9].

**Figure 4:**
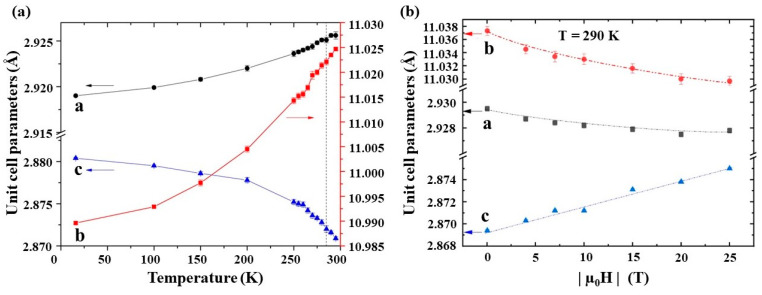
(**a**) The temperature dependence of lattice parameters, a, b and c, in zero magnetic field. The dotted line corresponds to the magnetic ordering temperature T_c_~290 K. Figure taken from [15]. (**b**) The magnetic field dependence of lattice parameters near T_c_. Figure taken from [22].

Subtle changes are noted in the lattice parameters observed near T_c_. In particular, the *a* and *b* lattice parameters show a positive thermal expansion, while *c* shows a negative thermal expansion. Figure 4a interatomic distances show a concomitant anisotropic evolution with temperature due to a combination of the lattice parameters and internal atomic coordinates, and it is deduced that the nearest-neighbor Fe–Fe interatomic distance within the (*ab*)-plane plays a central role in influencing the magnetofunctional response of these compounds [21]. Direct changes in the crystal structure of Fe_2_AlB_2_ under external magnetic fields have also been observed at high DC magnetic fields (up to ±25 T), and it was found that the unit cell parameter *c* increases while *a* and *b* decrease with increasing magnetic field (Figure 4b), with the most significant effect for the elongation of the *c*-axis in the vicinity of T_c_, which is consistent with computational calculations [15,22]. 

Despite the pronounced interrelationship between the lattice and the magnetic structure, it is intriguing that Fe_2_AlB_2_ demonstrates no hysteresis during magnetothermal cycling and that the latent heat is associated with the magnetic phase transformation near room temperature is rather modest [9,23]. Critical magnetic analysis using Arrot and Kouvel–Fisher plots (a valuable method to understand the exchange interaction and magnetic ordering in MCMs) suggests that the thermodynamic character of the FM → PM phase transition in Fe_2_AlB_2_ is second-order in nature [9,23,24]. Given these contradictions, it is surmised that the compound’s thermodynamic equilibrium state at room temperature is very close to a tricritical point between the first and second-order magnetic transitions [25]. This is advantageous because hysteresis represents the energy loss associated with the lag between the applied magnetic field and the material’s response [26]. In MCMs, hysteresis results in additional energy being lost as heat, which can increase the thermal load on the MHP system. This extra heat interferes with the system’s operation and necessitates other heat dissipation measures, further reducing the overall system efficiency.

## 3. Magnetofunctional Response of Fe_2_AlB_2_ and Its Compositional Variants

**Figure 5:**
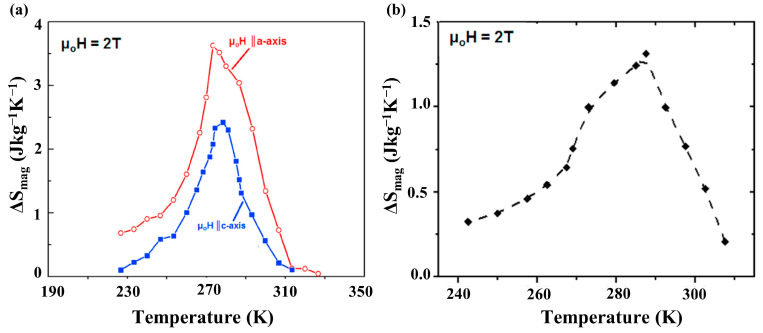
(**a**) Magnetic entropy change curves as a function of temperature measured along the *a*- and *c*-axis of Fe_2_AlB_2_ and (**b**) rotating magnetic entropy curves of the Fe_2_AlB_2_ crystallites at *μ*_0_*H* = 2 T. Figure taken from Ref. [17].

Interest in Fe_2_AlB_2_ in a magnetocaloric context was stimulated around a decade ago when Tan et al. reported an MCE corresponding to a magnetic entropy (∆S_mag_*)* and adiabatic temperature change (∆T_ad_) of 4.1 JK^−1^kg^−1^ and 1.8 K, respectively, in a magnetic field change of 0–2 T in the vicinity of the Curie temperature (T_C_ = 282 K) in a polycrystalline bulk sample [2]. Building on these results, an anisotropic magnetofunctional response was confirmed in Fe_2_AlB_2_ by measuring the response along the orthorhombic axes of a single crystallite. Temperature-dependent magnetic entropy change curves measured along the *a*- and *c*-axis of Fe_2_AlB_2_ (easy- and hard-axis of magnetization) indicate ∆S_mag_ values corresponding to 3.6 JK^−1^kg^−1^ and 2.4 JK^−1^kg^−1^, respectively, at μ_0_H_app_ = 2 T [17], resulting in a significant rotating magnetic entropy change of ΔS_rot_ = 1.3 J kg^−1^K^−1^ when the spin quantization vector is rotated from the hard *c*-axis to the easy *a*-axis direction, as shown in Figure 5a,b [17]. 

**Figure 6:**
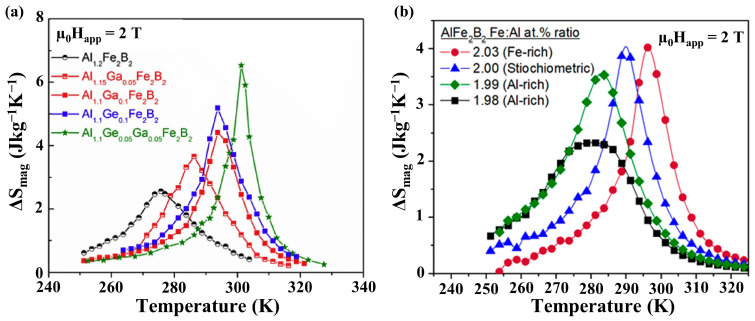
(**a**) Magnetic entropy change curves as a function of temperature for samples containing Fe-rich, stoichiometric, and Al-rich Al_(1−y)_Fe_(2+y)_B_2_ (y = −0.01–0.01). Figure taken from [7]; (**b**) magnetic entropy change curves of single phase samples of nominal composition, Al_1_._2−x_(Ga, Ge)_x_Fe_2_B_2_ (x < 0.1) at an applied magnetic field of μ_0_H_app_ = 2 T. Figure taken from [4].

The sensitivity of T_c_ and MCE to compositional variation has been the subject of many scientific publications (see Tables S1 and S2 in the Supplementary Material for a comprehensive summary), with most studies mainly focused on compositions where Fe in the lattice is substituted by other 3d transition-metal elements (V, Ti, Mn, Co, Ni) [12,27,28,29,30], Al is substituted by a post-transitional metal (Ga) or semi-metals (Ge, Si) [7,31,32] and B is substituted by the non-metal C [27,33]. Empirical trends indicate that the magnetofunctional response of Fe_2_AlB_2_ is typically driven by small changes in the crystal structure [30]. In particular, elemental compositions that change the *c*-axis length and the nearest neighbor Fe–Fe interatomic distance contribute the most significant chemical bonding effects to the alteration in the magnetic ordering and MCE response observed in its proximity [21]. Typically, chemically modified Fe_2_AlB_2_ compounds demonstrate a decrease in their overall magnetization, T_c_, and the associated MCE (∆S_mag_ and ∆T_ad_) relative to that of the parent compound, and their measured magnetic entropy change curves are usually broad, spanning 60–80 K. The only exception to these observations is noted in Ref. [7], wherein Fe_2_AlB_2_ alloys processed with small amounts of Ga and Ge displayed an almost two-fold increase in ∆S_mag_ values, as shown in Figure 6a. Intriguingly, the authors demonstrate that Ga and Ge were not detected in the Fe_2_AlB_2_ lattice using a variety of highly sensitive probes. Since Ga is intrinsically volatile, it is likely that during solidification from the melt, its loss is compensated by the formation of antisite defects wherein Fe occupies the Al site in the lattice, resulting in Fe-rich Fe_2_AlB_2_ compositions with improved MCE behavior relative to that of the stoichiometric compound. This hypothesis was subsequently confirmed when Lejeune et al. reported the growth of a single crystal of Fe_2_AlB_2_ in which the Fe and Al ratio was varied systematically along the direction of solidification from 1.94 to 2.06 [4]. Magnetic measurements indicate that ∆S_mag_ and T_c_ increase from 2.3 J/kg-K to 4.0 J/kg-K and 280 K to 315 K, respectively, as the Fe: Al ratio increases from 1.98 to 2.03, as shown in Figure 6b [4]. Thus, the upper limit of the MCE in Fe_2_AlB_2_ is constrained by the solid solubility limits of Al and Fe in the structure. 

## 4. Thermal and Electrical Transport Properties 

Understanding the transport properties of an MCM is essential for integrating magnetocaloric materials into practical heat pump systems [21]. While the MCE relies on the MCM’s ability to change temperature when exposed to a changing magnetic field, the specific heat capacity (C_p_) determines the amount of heat (Q) required to change the temperature (ΔT) of the material by a certain amount, according to the relation. $Q=mC_{p}∆T$, where m is the mass of the material. Maxwell’s relationships, as shown in Equation [3] and Equation (4) in Section 2(b), indicate that a lower C_p_ implies that a given amount of heat will result in a larger temperature change with minimal energy input, enhancing the MCE and making the cooling cycle more efficient. High thermal conductivity ensures rapid, uniform heat transfer, essential for efficient heat absorption and release during magnetization cycles [34]. High electrical resistivity minimizes eddy current losses and unwanted heating, enhancing energy efficiency [34]. Balancing these properties ensures effective thermal management, consistent performance, and long-term material stability, making MCMs viable for practical and efficient magnetic refrigeration systems. 

Table S3 in the Supplementary Material summarizes the transport properties of Fe_2_AlB_2_ [4,8,9,17]. The compounds demonstrate metallic behavior with a residual resistivity of 60 µΩ cm at 2K [9]. Temperature-dependent resistivity curves of Fe_2_AlB_2_ demonstrate a non-linear increase in resistivity with an increase in temperature and feature a kink in the proximity of T_c_, indicating a loss of spin disorder scattering due to a change in magnetic ordering. Room temperature values of electrical resistivity range from 1 × 10^−6^ to 4.5 × 10^−6^ Ohm-m, depending on the orientation and chemical composition of Fe_2_AlB_2_ single crystals [4]. The C_p_ of these compounds increases monotonically as a function of temperature except near the magnetic ordering temperature, where a peak is observed. The magnitude of the C_p_ peak is reported to be in the range of 110 J/mol-K to 147 J/mol-K, depending on the Fe:Al ratio in Fe_2_AlB_2_ compounds. The low C_p_ in Fe_2_AlB_2_ is accompanied by moderately high thermal conductivity (2.4 W/m-K to ~ 11.5 W/m-K in Al_1−y_Fe_2+y_B_2_ (−0.01 < y < 0.01) single crystals depending on the Fe:Al ratio) [4]. Using the time-domain thermoreflectance method, Lejeune et al. reported an anisotropic thermal conductivity behavior in Al-rich Fe_2_AlB_2_ polycrystals with values corresponding to κx = 4.7 ± 0.1 W/mK, κy = 4.4 ± 0.1 W/mK, and κz = 6.8 ± 0.3 W/mK [18]. Since AMRs should possess a significantly larger thermal conductivity in the direction of the heat transfer fluid’s path and low thermal conductivity in the transverse direction, it is conjectured that the anisotropic character of the thermal transport may be leveraged in an MHP device through the fabrication of crystallographically oriented AMR regenerators. 

## 5. Mechanical and Chemical Stability

Knowledge about the basic mechanical stability of MCM alloys is crucial for gauging the material’s ability to withstand repeated magnetization–demagnetization cycles without degradation. As a starting point in the evaluation of the mechanical robustness of Fe_2_AlB_2_, Table S4 in the Supplementary Material summarizes the nine independent elastic constants (*C_ij_*) of Fe_2_AlB_2_, while Table S5 provides the calculated values of bulk (*B*) [35,36,37,38,39,40,41,42], shear (*G*), and Young’s (*E*) moduli, as well as the Poisson ratio (*ν*) derived from the elastic constants. Following the requirements of mechanical continuum and stability in orthorhombic crystals, Fe_2_AlB_2_ meets the following criteria:

*C*_11_ > 0, *C*_22_ > 0, *C*_33_ > 0, C_44_ > 0, *C*_55_ > 0 (6)

(*C*_11_ + *C*_22_ − 2*C*_12_) > 0 (7)

(*C*_22_ + *C*_33_ − 2*C*_13_) > 0 (8)

*C*_11_ + *C*_22_ + *C*_33_ + 2(*C*_12_ + *C*_13_ + *C*_23_)] > 0 (9)

Consistent with the observation that strong B-B bonds are found along the a-axis (Figure 2a), the elastic constant *C*_11_ is larger than the other two principal elastic constants*, C_22_* and C*_33_*, implying that Fe_2_AlB_2_ is harder to compress along the a-axis than the b or c-axes [36]. The bulk modulus to shear modulus ratio, *B*/*G* of Fe_2_AlB_2_, is found to be less than 1.75, and therefore, following Pugh [43], it is categorized as a brittle material. The elastic anisotropic parameters of Fe_2_AlB_2_, as determined by the three parameters given below, are determined to be an orthorhombic system [40].

*A*_1_ = 4*C*_44_/(*C*_11_ + *C*_33_ − 2*C*_13_) (10)

*A*_2_ = 4*C*_55_/(*C*_22_ + *C*_33_ − 2*C*_23_) (11)

*A*_3_ = 4*C*_66_/(*C*_11_ + *C*_22_ − 2*C*_12_) (12)

The deviation of the anisotropic parameter *A*_i_ from unity denotes a material’s elastic anisotropy degree, and it is expected that it will exhibit variations in hardness depending on crystallographic orientation, influenced by the directional elastic constants and the behavior of the slip systems within the crystal structure. Reported values of Vickers hardness in Fe_2_AlB_2_ range from 11.6 to 14.6 GPa in single crystals to 9.3–10.7 GPa in polycrystals [35,36,37,38,39,40,41,42]. The hardness correlates to high brittleness, making the material more susceptible to crack initiation and propagation under stress, with low fracture toughness K1c ranging from 4.6 to 5.4 MPa·m^1/2^ [44,45,46,47].

The anisotropic elastic constants of Fe_2_AlB_2_ show a strong temperature dependence in the range of 0–1300 K, which is in alignment with the study of Verger et al. [48] that reports anisotropy in the coefficient of thermal expansion in Fe_2_AlB_2_ powders using in situ high-temperature X-ray diffraction measurements. As discussed in Section 2(b), all three lattice parameters of Fe_2_AlB_2_ show non-linear changes without any discontinuities, indicative of a second-order phase transition. The sharpest change in these parameters occurs at the Curie temperature, where the increase in the *a* and *b* lattice parameters is compensated by *a* decrease in the *c* lattice parameter, resulting in a negligible change in the unit cell volume—an important characteristic that will likely assist Fe_2_AlB_2_ in preserving its mechanical integrity during magnetic field cycling. Indeed, a simple proof-of-concept demonstration of this compound’s mechanical robustness may be found in Ref. [49], where Zhang et al. report no surface microcracks/flakes or degradation of magnetocaloric response in a suction-cast Fe_2_AlB_2_ sample subjected to 5 × 10^5^ cycles in and out of the 0.5 T magnetic field for a week. It is worth noting that this study also shows an overall decrease in the magnetization and the magnetocaloric response of Fe_2_AlB_2_ when it is exposed to water (often the heat transfer fluid in an MHP) for prolonged periods. The degradation in MCE is primarily due to surface oxidation, and it follows an approximate exponential-decay trend, which tends to stabilize with time. Though it has not been experimentally demonstrated yet, it may be conjectured that corrosion can weaken the Fe_2_AlB_2_’s structure over time, causing cracks, pits, or other microstructural defects that can ultimately result in mechanical failure. The corrosion mechanism of Fe_2_AlB_2_ remains largely unexplored, and this remains an open topic for investigation by the magnetocaloric community. 

## 6. Amenability to Shaping into Heat Exchange Structures

The mechanical properties of Fe_2_AlB_2_ guide the selection of machining parameters, tooling, forming processes, and overall manufacturing strategies during AMR fabrication. The ideal geometry of an AMR for efficient heat transfer needs a sizeable wetted surface, a small hydraulic diameter, and small solid structures to minimize thermal gradients within them. Since Fe_2_AlB_2_ alloys are brittle, it is most suitable for use in AMRS as packed particle beds (PB) [50]; however, the tortuous flow paths of this form will inevitably result in a steep increase in pressure drop with increasing flow rate, limiting operation to low frequencies. In recent years, the magnetocaloric community has been increasingly exploring fabricating AMRs with complex geometries (microchannels with a spatial dimension of ~100 μm [51,52], pin fin heat exchangers [53], Kagome lattice structures, porous foams, etc.) [51,54,55] using additive manufacturing processing schemes such as binder jetting, direct ink writing DIW, direct energy deposition DED, powder bed fusion PBF, and fused deposition modeling FDM [11,52,56,57,58,59,60,61,62,63,64,65,66,67,68,69,70,71,72,73,74,75].

**Figure 7:**
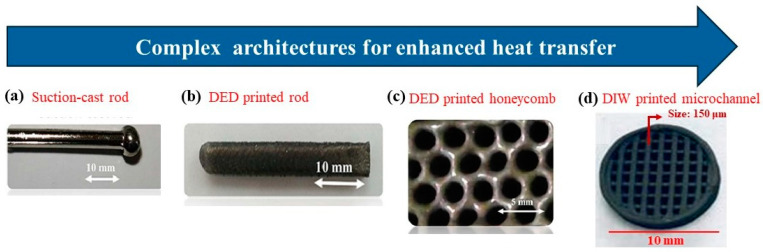
Advancements in metal AM technologies address the challenges of fabricating complex heat exchange structures with high surface-to-volume ratios and intricate geometries for magnetocaloric energy conversion. (**a**) Simple cylindrical rod of dia~3 mm obtained via suction casting [11]; (**b**) 3D-printed cylinder of dia~3 mm fabricated using DED [11]; (**c**) 3D-printed porous structure with thin-walled honeycombed channels with spatial resolution of ~3mm fabricated using DED [11]; (**d**) 3D-printed porous structure with thin-walled honeycombed channels with spatial resolution of ~150 μm fabricated using DIW technique [52].

**Figure 8:**
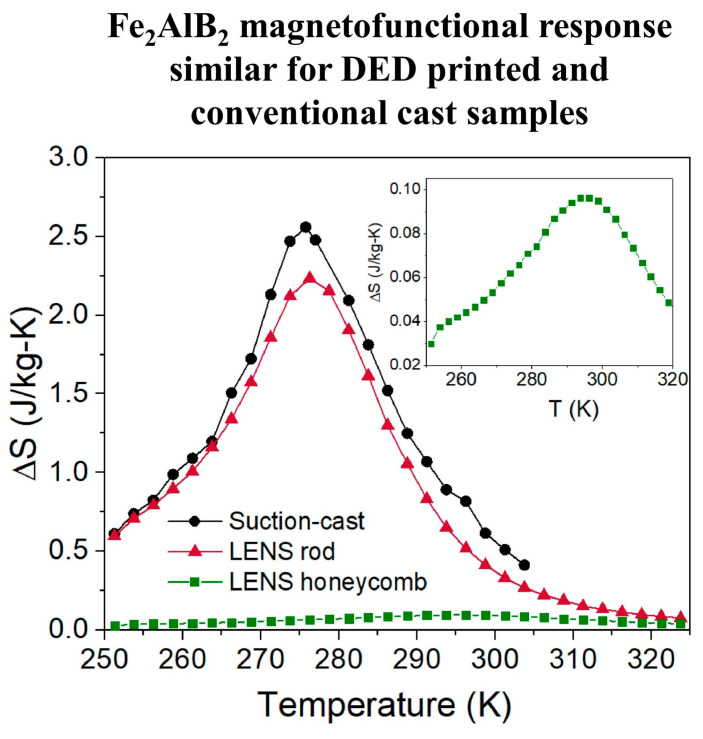
Magnetic entropy change curves as a function of temperature of the suction-cast and DED-processed cylindrical rods of nominal composition Fe_2_AlB_2_. No magnetocaloric response was observed in the 3D-printed channeled structure due to phase inhomogeneities. Figure taken from [11].

Ref. [11] reports on the feasibility of 3D printing Fe_2_AlB_2_ bulk samples with simple cylindrical shapes and complex honeycomb architectures via powder DED. Though good consistency was observed between the phase constitution and magnetofunctional behavior of the 3D-printed cylinders and bulk samples made by conventional suction-casting following a heat treatment at 1040 °C for three days (shown in Figure 7b, respectively), the honeycomb sample shown in Figure 7c exhibited a low-phase fraction of Fe_2_AlB_2_ and negligible magnetocaloric response—likely due to challenges associated with process parameter optimization during the printing process as shown in Figure 8. Compared to laser-based AM methods, DIW is an inexpensive, low-temperature processing method that does not involve melting and recrystallizing magnetic particles. Thus, it simply maintains the original particles’ compositional homogeneity and caloric properties. In Refs. [52,66,76], a DIW method is reported whose novelty lies in the initial material feedstock that consists of a magnetic ink comprising magnetocaloric particles (up to ~85 wt%) and sacrificial polymer binders dispersed in a multi-solvent system. While the solvents control the rheology of the ink, the polymer acts as a binding agent for the magnetic particles, facilitating the retention of the regenerator architecture. Subsequently, a low-temperature sintering process at 450 °C removes the binder, and a high-temperature sintering process at 1000 °C promotes grain growth and densification of the final finished part. Preliminary research efforts reported in Refs. [52,66] indicate that while Fe_2_AlB_2_ is amenable to shaping via DIW (Figure 7d), the 3D-printed test coupons showed poor structural integrity and magnetic response post-sintering due to the thermal instability of Fe_2_AlB_2_ when re-heated to temperatures above 600 °C and carbon contamination from the binder during heat treatment. 

## 7. Conclusions

This report provides insight into the fundamental origin of the near-room-temperature magnetocaloric response observed in the unique magnetic MAB compound Fe_2_AlB_2_ and provides a comprehensive summary of the magnetofunctional response, transport properties, and mechanical stability of this rare-earth-free material from the viewpoint of their potential application as AMRs in MHPs. Overall, it is surmised that this rare-earth-free MCM demonstrates a moderate magnetocaloric response, promising electrical and thermal transport properties, robust mechanical properties during magnetic field cycling, and reasonable chemical stability when exposed to a water-based heat-transfer fluid for prolonged periods. Like all layered MAB compounds, Fe_2_AlB_2_ demonstrates an anisotropic growth habit with pronounced anisotropic properties due to its orthorhombic crystal structure that features alternate Al layers and FeB chains. The magnetocrystalline anisotropy leads to a large magnitude of the magnetic entropy change when measured along the easy *a*-axis of Fe_2_AlB_2_ versus the *c*-axis, leading to an intriguing rotating magnetocaloric effect RMCE (*Δ*S_rot_ (1 T) ~ 1 J kg^−1^K− at ~290 K). Conversely, the measured thermal conductivity values are 40% larger along the *c*-axis than the corresponding a- and b-axes of Fe_2_AlB_2_. It is worthwhile to note that to date, significant rotating magnetocaloric effects near room temperature have only been reported in single crystals of rare-earth NdCo_5_ alloys and its Fe-doped compositional variants [77,78] (ΔS_rot_ (1 T) = 2.2–2.7 J Kg^−1^K^−1^ in the temperature range 275–300 K, depending of Fe concentration) and in an aligned polycrystalline alloy of NdCo_4_Al (ΔS_rot_ (1 T) *=* 1.9 J kg^−1^K^−1^ at 295 K) [79]. 

The anisotropic character of Fe_2_AlB_2_ has significant relevance for technological applications since the shape and orientation relative to the magnetic field can either enhance or diminish the magnetization of the AMR structure within an MHP device. For example, thin plates oriented along the magnetic field direction are more strongly magnetized than packed spheres due to internal demagnetization effects [80]. While it is relatively straightforward to synthesize Fe_2_AlB_2_ in bulk form using conventional casting, melt-spinning, and hot-pressing/reaction sintering techniques at temperatures as low as 1000 °C (a sheer advantage for large-scale bulk production and usage), it is hard to shape these materials into thin periodic walls or microchanneled heat exchange structures due to their inherent brittleness. Significant advancements in metal additive manufacturing (AM) technologies, particularly laser- and extrusion-based approaches, have emerged as we progress [11,52,66]. These methods effectively address the challenges of fabricating complex regenerator architectures with high surface-to-volume ratios and intricate geometries for magnetocaloric energy conversion. Moreover, through precise microstructure manipulation via rapid solidification in localized regions, AM can mitigate issues related to brittleness and phase inhomogeneities in MCMs. Only two publications have addressed the 3D printing of Fe_2_AlB_2_ into complex geometries, making it an undeveloped field for further exploration. 

Last, a quantitative comparison of Fe_2_AlB_2_ with Gd and La(Fe, Si)-based alloys is provided in Table S6 in the Supplementary Material. [2,4,7,8,9,14,17,18,25,81,82,83,84]. While the magnetocaloric response, heat transfer properties, and mechanical stability of Fe_2_AlB_2_ are found to be comparable to these commercially viable MCMs, a recently developed numerical model of the AMR cycle indicates that the cooling performance (in terms of temperature span between hot and cold sources ΔT_span_ and cooling capacity *Qc*) is significantly lower in Fe_2_AlB_2_ relative to Ga and La(Fe, Si)_13_. Despite these theoretical predictions, this magnetocaloric boride continues to interest the scientific community, mainly because it comprises abundant light elements and can be sustainably mass-produced for large-scale applications. With the further development of improved computational abilities (big data science) for prediction of new MCM compositions [85,86,87], it is anticipated that artificial intelligence (AI) computation techniques, such as machine learning, deep learning, and neural networks, will aid in the understanding, finding, and designing of new compositional variants of Fe_2_AlB_2_ with improved magnetocaloric properties (higher ΔT_ad_ values at lower magnetic fields), thus bridging the gap needed to make this MAB compound competitive with commercially viable state-of-the-art MCMs.

## Data Availability

Raw data pertaining to graphs and tables presented in this work may be provided upon request.

## Supplementary Materials

The following supporting information can be downloaded at: https://www.mdpi.com/article/10.3390/ma17163886/s1, Table S1: Effect of synthesis methods on the magnetocaloric properties of AlFe_2_B_2_ prepared using molten salt reactive sintering, compared with samples prepared using different synthesis methods; Table S2: Effect of compositional substitution/doping; Table S3: Transport Properties of Fe_2_AlB_2_; Table S4: Summary of *C_ij_* values of AlFe_2_B_2_ determined from computation calculations (unit: GPa); Table S5: The bulk modulus (*B* in GPa), shear modulus (*G* in GPa), Young’s modulus (*E* in GPa), Poison ratio (*µ* in GPa), *B/G* (Pugh ratio) and hardness (*H*v) of the AlFe_2_B_2_; Table S6: Transition temperature (Tc), heat capacity (Cp), thermal conductivity (k), and electrical resistivity (ρ) for Fe_2_AlB_2_ and other candidates for near-room temperature magnetic cooling. References [2,4,5,6,7,8,9,10,11,12,13,14,15,16,17,18,19,20,25,27,28,29,30,31,32,33,35,36,37,38,39,40,41,42,81,82,83,84,88,89,90] are cited in the supplementary materials.
