# Supplementary material for: Synthesis, Characterization, and Magnetocaloric Properties of the Ternary Boride Fe2AlB2 for Caloric Applications
Source: Materials (Basel). 2024 Aug 6;17(16):3886. doi: 10.3390/ma17163886 (PMC11355234; doi:10.3390/ma17163886)
Supplement: Supplementary file 1 [file materials-17-03886-s001.zip › materials-3107161-supplementary.pdf]

Supplementary Data:

Table 1: Effect of synthesis methods on the magnetocaloric properties of $AlFe_2B_2$ prepared using molten salt reactive sintering, compared with samples prepared using different synthesis methods.

Synthesis Method	Post Processing Methods				Magnetic Properties			Reference
	Al/Fe/B	Heat Treatment		Acid Etching	Tc (K)	Ms (emu/g)	ΔS(J/Kg^{-1}K^{-1})	
		Gas	Temp(K)					
**Powder Metallurgy**								
Molten salt sintering	1.2/2/2	Air	1273	N	300	62 at 1.8 K	3.8 at 2T	[1]
Reactive Sintering	1.2/2/2	Ar	1273	N	288	69 at 1.8 K	4.6 at 2T	[1]
Single step reactive hot pressing	1/2/2	Under Vacuum	1473	N	280	70 at 50 K	2.4 at 2T<br>5.3 at 5T	[2]
Spark Plasma Sintering	1/2/2	Under Vacuum	1183	N	274	6.2 at 5 K	3.1 at 2T<br>6.4 at 5T	[3]
Microwave Sintering	3/2.6/2	Under Vacuum	1173	Y	287	1.11 µB/Fe atom	3.31 at 2T<br>6.45 at 5T	[4]
**Melt Based Processes**								
Arc Melting	3/2/2	Under Vacuum	1173	Y	282	1.15 µB/Fe atom	4.4 at 2T<br>7.3 at 5T	[5]
Arc Melting	1.5/1/1	Ar	1073	N	320	0.95 µB/Fe atom	-	[6]
Arc Melting	3/1/1	Under Vacuum	1173	Y	285	0.9 µB/Fe atom	1.3 at 1T<br>4.5 at 5T	[7]
Arc Melting	3/2/1	Under Vacuum	1173	Y	299	-	-	[8]
Arc Melting	(2-3)/2/2	Under Vacuum	1073	Y	290	-	-	[3]

Arc Melting	3/2/2	-	-	Y	285	78 at 50 K	2.4-3.6 (hard/easy axis) at 2 T	[9]
Arc Melting	3/2/2	-	-	Y	286	0.7 μB/Fe atom	-	[10]
Arc Melting	2/2/2	-	-	N	290	55.8 at 100 K	1.85 at 2T<br>4 at 5T	[11]
Arc Melting	2/2/2	-	-	Y	294	72.3 at 100 K	3.07 at 2T<br>6.49 at 5T	[11]
Arc Melting	3/2/2	-	1173	Y	303	91.35 at 5 K	3.4 at 2T<br>7.2 at 5T	[12]
Arc Melting	3/2.6/2	Under Vacuum	1173	Y	286	1.23 μB/Fe atom	3.75 at 2T<br>7.21 at 5T	[4]
Arc Melting	1/2/2	Under Vacuum	1178	N	278	-	6.3 at 7T	[13]
Arc Melting	1.2/2/2	Ar	1323	N	-	-	-	[14]
Arc Melting/Suction casting	1.2/2/2	Ar	1313	N	272	78 emu/g at 50 K	2.7 at 2T	[15]
Suction cast	1/2/2	Ar	1313	N	293	76.7 at 50 K	-	[16]
Suction cast	1.2/2/2	Ar	1313	N	282	68.7 at 50 K	-	[16]
Ga Flux<br>Stoichiometric	1.5/1.8/2	Under Vacuum	1173	Y	307	1.03 μB/Fe atom	4.1 at 2T<br>7.7 at 5T	[5]
Al Flux	5/3/2	Ar	1473	Y	274	2.40 μB/f.u.	3.78 at 2T<br>4.87 at 3T	[17]
Al Flux	0/1/2/5(Al):1(AlFe2B2)	Ar	1673	Y	-	-	-	[3]
R-F Melting	7/7/6	Ar	1873	Y	293	1.28 μB/Fe atom	3.1 for 2T	[4]

							6.75 for 5T	
R-F Melting	3/2.6/2	Ar	1873	Y	287 K	1.13 μB/Fe atom	3.38 for 2T<br>6.47 for 5T	[4]
Induction Melting	1.2/2/2	Under Vacuum	1173	N	302	79.9	7.2 for 5T	[18]
Induction Melting	1.2/2/2	-	-	N	300	-	5.1 for 5T	[18]
Induction Melting	1.8/2/2	Under Vacuum	1173	N	285	69.9	4.8 for 5T	[18]
Induction Melting	1.8/2/2	-	-	N	268	-	1.8 for 5T	[18]
Direct Energy Deposition	4/2/4	Ar	1313	N	284	65.2 at 50 K	2.2 for 2T	[19]

**Table 2:** Effect of compositional substitution/doping					
**Nominal Composition**	**Compositional Variants**	**Tc (K)**	**Δ S (J/Kg.K)**	**Δ T (K)**	**References**
**Substitution of Al with Ga, Ge and Si**					
$Al_{1.15}Ga_{0.05}Fe_2B_2$	Ga	282	3.67 at 2 T	1.26 at 2 T	[15]
$Al_{1.1}Ga_{0.1}Fe_2B_2$	Ga	290	4.42 at 2 T	1.39 at 2 T	[15]
$Al_{1.15}Ga_{0.05}Ga_{0.05}Fe_2B_2$	Ga	294	6.51 at 2 T	2.19 at 2 T	[15]
$Al_{1.1}Ge_{0.1}Fe_2B_2$	Ge	291	5.18 at 2 T	1.74 at 2 T	[15]
$Al_{0.9}Ga_{0.1}Fe_2B_2$	Ga	300	6 at 5 T and 7.5 at 7 T	-	[20]
$Al_{0.8}Ga_{0.2}Fe_2B_2$	Ga	304	6 at 5 T and 7.5 at 7 T	-	[20]
$Al_{0.9}Si_{0.1}Fe_2B_2$	Si	294	4.9 at 5 T and 6.3 at 7 T	-	[20]
$Al_{0.8}Si_{0.2}Fe_2B_2$	Si	302	4.9 at 5 T and 6.3 at 7 T	-	[20]
$Al_{0.7}Si_{0.3}Fe_2B_2$	Si	306	4.6 at 5 T and 5.6 at 7 T	-	[20]
$Al_{0.87}Si_{0.15}Fe_2B_2$	Si	305	3.2 at 2 T and 6.6 at 5 T	-	[21]

$Al_{0.89}Si_{0.15}Fe_2B_2$	Si	297	2.1 at 2 T and 4.2 at 5 T	-	[21]
**Substitution of Fe with Ti, V, Mn and Ni**					
$AlFe_{1.9}V_{0.1}B_2$	V	280 at 0.05 T	4 at 7 T	-	[22]
$AlFe_{1.85}V_{0.15}B_2$	V	284 at 0.05 T	2.8 at 7 T	-	[22]
$AlFe_{1.9}Ti_{0.1}B_2$	Ti	282 at 0.05 T	4.2 at 7 T	-	[22]
$AlFe_{1.85}Ti_{0.15}B_2$	Ti	285 at 0.05 T	2.9 at 7 T	-	[22]
$AlFe_{1.975}Co_{0.025}B_2$	Co	272	2.78 at 2 T and 6 at 5 T	-	[3]
$AlFe_{1.95}Co_{0.05}B_2$	Co	269		-	[3]
$AlFe_{1.925}Co_{0.075}B_2$	Co	262	2.86 at 2 T and 5.9 at 5 T	-	[3]
$AlFe_{1.9}Co_{0.1}B_2$	Co	260		-	[3]
$AlFe_{1.875}Co_{0.125}B_2$	Co	255	2.7 at 2 T and 5.8 at 5 T	-	[3]
$AlFe_{1.85}Co_{0.15}B_2$	Co	260		-	[3]
$AlFe_{1.825}Co_{0.175}B_2$	Co	245		-	[3]
$AlFe_{1.8}Co_{0.2}B_2$	Co	234		-	[3]
$AlFe_{1.775}Co_{0.225}B_2$	Co	259		-	[3]
$AlFe_{1.75}Co_{0.25}B_2$	Co	224		-	[3]
$AlFe_{1.725}Co_{0.275}B_2$	Co	215		-	[3]
$AlFe_{1.7}Co_{0.3}B_2$	Co	205		-	[3]
$AlFe_{1.6}Mn_{0.4}B_2$	Mn	242		-	[23]
$AlFe_{1.35}Mn_{0.65}B_2$	Mn	220		-	[23]
$AlFe_{1.2}Mn_{0.8}B_2$	Mn	188		-	[23]
$AlFeMnB_2$	Mn	119		-	[23]
$AlFe_{0.8}Mn_{1.2}B_2$	Mn	43		-	[23]
$AlFe_{0.4}Mn_{1.6}B_2$	Mn			-	[23]
$AlFe_{1.9}Mn_{0.1}B_2$	Mn	288	2.53 at 2 T and 5.45 at 5 T	-	[24]
$Al_{1.2}(Fe_{0.95}Ni_{0.05})_2B_2$	Ni	288		-	[25]
$Al_{1.2}(Fe_{0.9}Ni_{0.1})_2B_2$	Ni	291		-	[25]
**Substitution of B with C and (Ge,Ga)**					
$Al_{1.2}Fe_2(B_{0.9}C_{0.1})_2$	C	305		-	[25]
$Fe_2Al_{1.1}B_2Ge_{0.05}Ga_{0.05}$	GeGa	305	3.5 at 2 T and 6.7 at 5 T	1.1 at 1.93 T	[26]
$AlFe_2B_{1.9}C_{0.1}$	C	290 at 0.05 T	3.9 at 7 T	-	[22]
$AlFe_2B_{1.8}C_{0.2}$	C	295 at 0.05 T	2.5 at 7 T	-	[22]

**Table 3.** Transport Properties of Fe_2AlB_2		
**Figure of Merit**	**Single Crystal**	**Polycrystal**
**Heat capacity Cp (J/mol-K)**	117-147	115
**Thermal conductivity k (W/m-K)**	2.4 – 11.5	Kx = 4.7, Ky = 4.4, Kz = 6.8
**Electrical resistivity**	1 x 10-6 – 4.5 x 10-6 Ω-m	1.6 x 10^{-6} – 2.1 x 10^{-6} Ω-m
**References**	[16,17,27]	[9,16]

**Table 4.** Summary of C_{ij} values of $AlFe_2B_2$ determined from computation calculations (unit: GPa)									
**Material Compound**	***C*11**	***C*22**	***C*33**	***C*44**	***C*55**	***C*66**	***C*12**	***C*13**	***C*23**	**References**
Fe_2AlB_2	389.1	433.2	348.7	171.5	137.5	141.2	138.7	128.1	118.3	[28]
Fe_2AlB_2	447.0	402.7	334.6	140.2	166.3	156.2	170.1	133.9	156.4	[29]
Fe_2AlB_2	397	344	276	131	166	131	128	86	105	[30]
Fe_2AlB_2 Exp*	419	367	307	127	164	129	133	118	118	[30]
Fe_2AlB_2	455.7	404.8	318.2	148.9	179.7	160.8	184.0	126.2	179.6	[31]
Fe_2AlB_2	456.94	404.37	344.29	148.23	179.59	159.49	183.10	124.81	162.83	[32]
Fe_2AlB_2	419.9	372.0	319.5	130.6	168.0	131.0	152.5	117.6	134.5	[33]
Fe_2AlB_2	415.40	370.88	308.83	130.76	167.74	130.37	152.06	113.57	133.91	[34]
Fe_2AlB_2	426.0	393.0	354.3	124.0	157.6	125.2	152.3	140.0	130.1	[35]

Exp* indicates that the crystal lattice has been corrected with experimental values.

**Table 5.** The bulk modulus (*B* in GPa), shear modulus (*G* in GPa), Young's modulus (*E* in GPa), Poison ratio (μ in GPa), *B/G* (Pugh ratio) and hardness (*H*v) of the $AlFe_2B_2$							
**Material Compound**	B_{HVR}	G_{HVR}	***E***	μ	***B/G***	***H*v**	**References**
Fe_2AlB_2	214.7	141.4	-	0.23	1.52	-	[28]
Fe_2AlB_2	231.8	138.8	347.2	0.25	1.67	20.5	[29]
Fe_2AlB_2 (PBE)	181	130	315	0.21	-	-	[30]
Experimental	162.4	107.3	264.5	0.229	51	-	[30]

Fe_2AlB_2 Exp (PBE)	201	131	323	0.233	-	-	[30]
Fe_2AlB_2	236.1	139.4	349.3	0.25	1.69	16.4	[31]
Fe_2AlB_2	236.15	143.70	308.79	0.28	1.64	17.51	[32]
Fe_2AlB_2	211.65	131.37	326.56	0.2429	1.6111	16.86	[33]
Fe_2AlB_2	208.54	130.28	323.49	0.241	1.6006	16.92	[34]

Exp* indicates that the crystal lattice has been corrected with experimental values.

Table 6. Transition temperature (Tc), heat capacity (Cp), thermal conductivity (k), and electrical resistivity (ρ) for Fe_2AlB_2 and other candidates for near-room temperature magnetic cooling.

**Material**	**Tc (K)**	**Cp (J/mol-K)**	**k (W/m-K)**	**(Ω-m)**	**References**
Fe_2AlB_2	280-320	110-147	2.4 – 11.5	1 x 10^{-6} – 4.5 x 10^{-6} Ω-m	[5,6,9,15–17,27,36,37]
Gd	270-310	50 - 100	10 - 14	-	[37,38]
$Gd_5(Si,Ge)_4$	150-335	90	6	24 x 10^{-6} Ω-m	[37,39,40]
$La(Fe,Si)_{13}$	198 -356	900	10 - 15	1.5 × 10^{-6} Ω·m	[37,41]

References:

1. Sharma, V.; Dey, M.; Duong, A.; Gupta, S.; Barua, R. Magnetofunctional Response of AlFe2B2 Powders Synthesized in Open Air via Molten Salt Shielded/Sintering Method. *MRS Commun.* **2023**, *13*, 574–580, doi:10.1557/s43579-023-00385-8.

2. Bennett, S.P.; Kota, S.; ElBidweihy, H.; Parker, J.F.; Hanner, L.A.; Finkel, P.; Barsoum, M.W. Magnetic and Magnetocaloric Properties of Fe2AlB2 Synthesized by Single-Step Reactive Hot Pressing. *Scr. Mater.* **2020**, *188*, 244–248, doi:10.1016/j.scriptamat.2020.07.042.

3. Hirt, S.; Yuan, F.; Mozharivskyj, Y.; Hillebrecht, H. AlFe2-XCoxB2 (x = 0-0.30): TC Tuning through Co Substitution for a Promising Magnetocaloric Material Realized by Spark Plasma Sintering. *Inorg. Chem.* **2016**, *55*, 9677–9684, doi:10.1021/acs.inorgchem.6b01467.

4. Mann, D.K.; Wang, Y.X.; Marks, J.D.; Strouse, G.F.; Shatruk, M. Microwave Synthesis and Magnetocaloric Effect in AlFe2B2. *Inorg. Chem.* **2020**, *59*, 12625–12631, doi:10.1021/acs.inorgchem.0c01731.

5. Tan, X.; Chai, P.; Thompson, C.M.; Shatruk, M. ChemInform Abstract: Magnetocaloric Effect in AlFe 2 B 2 : Toward Magnetic Refrigerants from Earth-Abundant Elements. . *ChemInform* **2013**, *44*, doi:10.1002/chin.201341016.

6. Elmassalami, M.; Oliveira, D.D.S.; Takeya, H. On the Ferromagnetism of AlFe2B2. *J. Magn. Magn. Mater.* **2011**, *323*, 2133–2136, doi:10.1016/j.jmmm.2011.03.008.

7. Cedervall, J.; Andersson, M.S.; Sarkar, T.; Delczeg-Czirjak, E.K.; Bergqvist, L.; Hansen, T.C.; Beran, P.; Nordblad, P.; Sahlberg, M. Magnetic Structure of the Magnetocaloric Compound AlFe2B2. *J. Alloys Compd.* **2016**, *664*, 784–791, doi:10.1016/j.jallcom.2015.12.111.

8. Cedervall, J.; Häggström, L.; Ericsson, T.; Sahlberg, M. Mössbauer Study of the Magnetocaloric Compound AlFe2 B 2. *Hyperfine Interact.* **2016**, *237*, doi:10.1007/s10751-016-1223-7.

9. Barua, R.; Lejeune, B.T.; Ke, L.; Hadjipanayis, G.; Levin, E.M.; McCallum, R.W.; Kramer, M.J.; Lewis, L.H. Anisotropic Magnetocaloric Response in AlFe2B2. *J. Alloys Compd.* **2018**, *745*, 505–512, doi:10.1016/j.jallcom.2018.02.205.

10. Ali, T.; Khan, M.N.; Ahmed, E.; Ali, A. Phase Analysis of AlFe2B2 by Synchrotron X-Ray Diffraction, Magnetic and Mössbauer Studies. *Prog. Nat. Sci. Mater. Int.* **2017**, *27*, 251–256, doi:10.1016/j.pnsc.2017.03.007.

11. Lee, J.W.; Song, M.S.; Cho, K.K.; Cho, B.K.; Nam, C. Magnetocaloric Properties of AlFe2B2 Including Paramagnetic Impurities of Al13Fe4. *J. Korean Phys. Soc.* **2018**, *73*, 1555–1560, doi:10.3938/jkps.73.1555.

12. Du, Q.; Chen, G.; Yang, W.; Song, Z.; Hua, M.; Du, H.; Wang, C.; Liu, S.; Han, J.; Zhang, Y.; et al. Magnetic Properties of AlFe2b2 and CeMn2Si2 Synthesized by Melt Spinning of Stoichiometric Compositions. *Jpn. J. Appl. Phys.* **2015**, *54*, doi:10.7567/JJAP.54.053003.

13. Wang, S.; Liu, P.; Chen, J.; Cui, W. Magnetostriction and Heat-Capacity Study on the Metamagnetic Phase Transition of Dy2In1-XAlxalloys. *AIP Adv.* **2022**, *12*, 1–5, doi:10.1063/9.0000361.

14. Sivaprahasam, D.; Kumar, A.; Jayachandran, B.; Gopalan, R. Thermoelectric Properties of Sb Doped AlFe$_2$B$_2$. **2022**, 1–5.

15. Barua, R.; Lejeune, B.T.; Jensen, B.A.; Ke, L.; McCallum, R.W.; Kramer, M.J.; Lewis, L.H. Enhanced Room-Temperature Magnetocaloric Effect and Tunable Magnetic Response in Ga-and Ge-Substituted AlFe2B2. *J. Alloys Compd.* **2019**, *777*, 1030–1038, doi:10.1016/j.jallcom.2018.10.206.

16. Levin, E.M.; Jensen, B.A.; Barua, R.; Lejeune, B.; Howard, A.; McCallum, R.W.; Kramer, M.J.; Lewis, L.H. Effects of Al Content and Annealing on the Phases Formation, Lattice Parameters, and Magnetization of A Lx F E2 B2 (X=1.0,1.1,1.2) Alloys. *Phys. Rev. Mater.* **2018**, *2*, 0–30, doi:10.1103/PhysRevMaterials.2.034403.

17. Lamichhane, T.N.; Xiang, L.; Lin, Q.; Pandey, T.; Parker, D.S.; Kim, T.H.; Zhou, L.; Kramer, M.J.; Bud'Ko, S.L.; Canfield, P.C. Magnetic Properties of Single Crystalline Itinerant Ferromagnet AlFe2 B2. *Phys. Rev. Mater.* **2018**, *2*, 1–12, doi:10.1103/PhysRevMaterials.2.084408.

18. Han, K.; Li, M.; Gao, M.; Wang, X.; Huo, J.; Wang, J.Q. Improved Magnetocaloric Effects in AlFe2B2 Intermetallics through the Enhancement of Magnetoelastic Coupling. *J. Alloys Compd.* **2022**, *908*, 164663, doi:10.1016/j.jallcom.2022.164663.

19. Lejeune, B.T.; Barua, R.; Simsek, E.; McCallum, R.W.; Ott, R.T.; Kramer, M.J.; Lewis, L.H. Towards Additive Manufacturing of Magnetocaloric Working Materials. *Materialia* **2021**, *16*, 101071, doi:10.1016/j.mtla.2021.101071.

20. Zhang, Z.; Yao, G.; Zhang, L.; Jia, P.; Fu, X.; Cui, W.; Wang, Q. Magnetic Phase Transition and Room-Temperature Magnetocaloric Effects in (Al,M)Fe 2 B 2 (M = Si, Ga) Compounds. *J. Magn. Magn. Mater.* **2019**, *484*, 154–158, doi:10.1016/j.jmmm.2019.03.082.

21. Stillwell, K.; Kramer, N.; Birch, B.; Reese, B.; Pathak, A.K.; Khan, M. The Magnetic and Magnetocaloric Properties of Al-Rich Al0.85+ XSi0.15Fe2B2compounds Prepared by Drop-Casting. *AIP Adv.* **2023**, *13*, 0–5, doi:10.1063/9.0000538.

22. Wang, S.; Liu, P.; Chen, J.; Cui, W. Substitution Effects on the Magnetic Phase Transition and Magnetocaloric Effects in Nanolaminated AlFe2B2alloys. *AIP Adv.* **2022**, *12*, doi:10.1063/9.0000362.

23. Chai, P.; Stoian, S.A.; Tan, X.; Dube, P.A.; Shatruk, M. Investigation of Magnetic Properties and Electronic Structure of Layered-Structure Borides AlT2B2 (T=Fe, Mn, Cr) and AlFe2-XMnxB2. *J. Solid State Chem.* **2015**, *224*, 52–61, doi:10.1016/j.jssc.2014.04.027.

24. Du, Q.; Chen, G.; Yang, W.; Wei, J.; Hua, M.; Du, H.; Wang, C.; Liu, S.; Han, J.; Zhang, Y.; et al. Magnetic Frustration and Magnetocaloric Effect in AlFe2-XMnxB2 (x = 0-0.5) Ribbons. *J. Phys. D. Appl. Phys.* **2015**, *48*, doi:10.1088/0022-3727/48/33/335001.

25. Lejeune, B.T.; Jensen, B.A.; Barua, R.; Stonkevitch, E.; McCallum, R.W.; Kramer, M.J.; Lewis, L.H. Lattice-Driven Magnetic Transitions in Al(Fe,T) 2 X 2 Compounds. *J. Magn. Magn. Mater.* **2019**, *481*, 262–267, doi:10.1016/j.jmmm.2019.03.026.

26. Al, F.; Ge, B.; Beckmann, B.; El-melegy, T.A.; Koch, D.; Wiedwald, U.; Farle, M. Reactive Single-Step Hot-Pressing and Magnetocaloric Performance of Polycrystalline.

27. Lejeune, B.T.; Schlagel, D.L.; Jensen, B.A.; Lograsso, T.A.; Kramer, M.J.; Lewis, L.H. Effects of Al and Fe Solubility on the Magnetofunctional Properties of AlFe2 B2. *Phys. Rev. Mater.* **2019**, *3*, doi:10.1103/PhysRevMaterials.3.094411.

28. Cheng, Y.; Lv, Z.L.; Chen, X.R.; Cai, L.C. Structural, Electronic and Elastic Properties of AlFe2B 2: First-Principles Study. *Comput. Mater. Sci.* **2014**, *92*, 253–257, doi:10.1016/j.commatsci.2014.05.048.

29. Kádas, K.; Iuşan, D.; Hellsvik, J.; Cedervall, J.; Berastegui, P.; Sahlberg, M.; Jansson, U.; Eriksson, O. AlM2B2 (M = Cr, Mn, Fe, Co, Ni): A Group of Nanolaminated Materials. *J. Phys. Condens. Matter* **2017**, *29*, doi:10.1088/1361-648X/aa602a.

30. Qin, K.; Qi, X.; Gao, J.; He, X.; Yin, H.; Yin, W.; Song, G.; Zheng, Y.; Bai, Y. *Improving the Density Functional Theory Prediction Accuracy for Elastic Moduli and Thermal Expansion of MoAlB, Fe2AlB2, and Mn2AlB2*; 2024; Vol. 107; ISBN 8645186403.

31. Atalay, A.S.; Derin, B. Mechanical Effects of Cr and V Substitutions in AlFe2B2 by First-Principles Calculations. *Comput. Mater. Sci.* **2024**, *239*, 112960, doi:10.1016/j.commatsci.2024.112960.

32. Liu, Y.Z.; Sun, L.; Zheng, B.C.; Yi, Y.L.; Zhai, W.Y.; Peng, J.H.; Li, W. Anisotropic Elastic, Thermal Properties and Electronic Structures of M2AlB2 (M=Fe, Cr, and Mn) Layer Structure Ceramics. *Ceram. Int.* **2021**, *47*, 1421–1428, doi:10.1016/j.ceramint.2020.08.266.

33. Wang, R.; Tao, X.; Ouyang, Y.; Chen, H.; Peng, Q. Suggest a New Approach to Fabricate AlFe2B2. *Comput. Mater. Sci.* **2020**, *171*, 109239, doi:10.1016/j.commatsci.2019.109239.

34. Wang, Q.; Ding, H.; Tian, F. Temperature Dependent Mechanical Properties of MAB Phase Fe2AlB2. *Comput. Condens. Matter* **2023**, *34*, e00786, doi:10.1016/j.cocom.2023.e00786.

35. Aydin, S.; Şimşek, M. Pressure-Induced Magnetic Phase Transitions of Intermetallic Fe2AlB2. *J. Magn. Magn. Mater.* **2020**, *502*, doi:10.1016/j.jmmm.2020.166453.

36. Lejeune, B.T.; Barua, R.; Mudryk, Y.; Kramer, M.J.; McCallum, R.W.; Pecharsky, V.; Lewis, L.H. Borderline First-Order Magnetic Phase Transition in AlFe2B2. *J. Alloys Compd.* **2021**, *886*, 161150, doi:10.1016/j.jallcom.2021.161150.

37. Lejeune, B.T.; Du, X.; Barua, R.; Zhao, J.C.; Lewis, L.H. Anisotropic Thermal Conductivity of Magnetocaloric AlFe2B2. *Materialia* **2018**, *1*, 150–154, doi:10.1016/j.mtla.2018.05.011.

38. Sousa, J.B.; Pinto, R.P.; Amado, M.M.; Pinheiro, M.F.; Moreira, J.M.; Braga, M.E. Critical Behaviour of the Thermal Conductivity Near the Curie Point of Gadolinium. *J.*

Phys. Paris **1980**, *41*, 573–578, doi:10.1051/jphys:01980004106057300.

39. Szade, J.; Skorek, G. Electronic Structure and Magnetism of Gds (Si , Ge) 4 Compounds. **1999**, *197*, 699–700.

40. Brück, E. Developments in Magnetocaloric Refrigeration. *J. Phys. D. Appl. Phys.* **2005**, *38*, doi:10.1088/0022-3727/38/23/R01.

41. Palstra, T.T.M.; Mydosh, J.A.; Nieuwenhuys, G.J.; van der Kraan, A.M.; Buschow, K.H.J. Study of the Critical Behaviour of the Magnetization and Electrical Resistivity in Cubic La(Fe, Si)13 Compounds. *J. Magn. Magn. Mater.* **1983**, *36*, 290–296, doi:10.1016/0304-8853(83)90128-2.
